# The Wealth of Dentists

**Published:** 1890-04

**Authors:** C. M. Wright


### The Wealth of Dentists.

BY PROF. C. M. WRIGHT, D.D.S.

Read before the Mississippi Valley Dental Society, March 6th, 1890.

*Gentlemen:*—When, in answer to the request of your executive committee, I agreed to write a paper, and gave the subject as the "Wealth of Dentists," a great number of beautiful, fantastic, instructive and valuable ideas were chasing each other through the gray matter situated in the cortex of my cerebri. When I seized my pen and prepared to find some of these aforesaid ideas, I found them very slippery in morphological characteristics and extremely difficult to catch. I feel like a man trying to eat oysters with a toothpick, or peas with a two-pronged fork. Nevertheless, having promised the committee, I have faithfully persisted and hope to serve up a few mutilated ideas on the subject advertised.

In considering the wealth of a nation, some writers have not only patiently enumerated the various possessions of the nation of a purely materialistic nature—such as surplus of gold and silver in the vaults of the treasury, excess of income over output, of revenue duties, interests on money loaned to railroads and foreign countries, increasing value of land, etc., etc.—but have also taken an account of the more intangible, but none the less more real variable conditions, such as the climate of the country, the health and morality of the inhabitants of the country, the increasing intelligence of the various communities. In other words, these writers have placed the morality, the intellectual growth and the health of the people—the physical and mental prosperity of the inhabitants themselves—as items to be added up with the grosser material prosperity of the country.

I am glad that writers have taken this course—for in writing about the wealth of a class of men such as the dentists, I find that if I should be compelled to confine myself to the purely material view, and refer only to dollars, houses, stocks, incomes, and other such worldly possessions, my essay would be complete in a very few words. In fact, the subject would hardly be worth writing about if only this narrow view presented itself. While in common with others who have studied the question of wealth as a possession by nations or individuals, I shall not neglect the question of the material possession of the dentist, I nevertheless think it fortunate that I have the privilege of referring to the moral and mental possessions of this class about which I am writing. The individual is in many respects like the nation. The law good for the individual is the law good for the nation. The economy good for the individual is the economy good for the nation. The principles of right and justice good for one are good for the other, and so on in a number of propositions. Scientists have been pleased to reason in this way in regard to laws of nature, and profess to see in the gradual evolution of the individual from a microscopic mass of protoplasm to a complete organism such as man, the law of all existing things—as true of a plant as of a planet. Not to devote any more time to preliminary observations, let us plunge *in medias res*, by stating that the dentist is a man of capital—but his capital is of a peculiar kind. It does not consist of money in the form of gold or silver, or negotiable bills, nor of deeds to property in lands or houses which are transferable. His capital, however, has a money value only in the sense that a part of it, at least, has cost money. His capital, in part, can be computed in money, when we consider the actual cost in money of the instruction he has paid for—the cost of his living for the periods of time included in his years of pupilage and in his years of waiting for practice; also, the cost of office rent, furniture, his instruments, implements and appliances. In the settling of his estate, however, much of his capital could not be set down as assets. With the exception of the second-hand value of furniture and implements the other costly capital can not be made available as assets. How often this is brought to our notice after the death of one of our brothers. His assets amount to a few hundreds or at best a very few thousands of dollars, while his working capital before death could be easily and fairly estimated as a non-transferable capital of many thousands of dollars. The reputed skill, the moral integrity, the business capacity, the persistent efforts, the "personal practice," of the man in his prime are practically worth, in some cases, hundreds of thousands of dollars placed at interest at six per cent. per annum. The man owning $100,000 in money frequently works hard to make this money earn for him an income of six per cent., or $6,000. The dentist earning $6,000 from practice, is then, in one sense, worth to his family $100,000. Twelve thousand a year at six per cent. equals $200,000.

But the $200,000 is only a life interest. It is like a gas well which is profitable as long as it flows, and the flow may stop or decrease in quantity at any time. Another point in regard to a dentist's capital (shall we say floating capital?) is that it is at its maximum for about a quarter of a century, at best, or say from thirty to sixty years of age. Few men before thirty years of age have an income of much significance, and by sixty years of age, the work of the average dentist as far as an income-gatherer is over. So that the material wealth of the dentist consists of an *income of money*, for, let us say, thirty years, which income during this period, is sufficient for himself and family, enabling them to maintain a home establishment, affording great comfort and a degree of what we may call quiet elegance. The most successful and business-like dentists, by the exercise of some financial skill, and considerable prudence in the way of expenditure during the period of the thirty years of paying practice, become real owners of the homes of their families, and I use the term *real* owners, because of the frequent and uncomfortable mortages casting clouds over the clear title of many of these elegant homes believed to be owned by dentists in good practice.

Besides the good income—that is the income which affords the comforts and elegancies of the 19th century, in a scale of society as far removed from the lowest, as it is from the highest grades, the successful dentist is generally the possessor of life insurance policies valued at from 5,000 to 30,000 or 40,000 dollars—which his income permits him to maintain and which form the most important part of the effects of the dead dentist who has been a successful and prudent dentist during his years of activity. This is all that I can say in regard to the material wealth of the dentist.

What are some of the other possessions of the dentist which can be added up in the items to his credit? First, I shall mention *morality*. Dentists, as a class, possess this noble quality in a high degree. Morality is a cultivated nerve centre, implanted in the highest portion of the brain, from which two sets of fibres radiate—the inhibitory and the accelleratory. The numerous inhibitory fibres pass out from the centre and extend to a great variety of terminal points in the brain and to other centres, checking and controlling as with a rein a great variety of thoughts and actions. The accelleratory fibres, on the contrary, extend to the centres of noble and true emotions, impelling them to activity, and "good actions" are the result. This quality of morality is not equally strong in the young. It is, of course, influenced by heredity, as are other qualities, but it is capable of cultivation—it is developed by increased activity of its function, and affected by sundry conditions. The orthodox religious sentiments of the day do not appear to be very powerful irritants of this centre, for religious convictions and enthusiasm do not always walk hand in hand with high morality. The daily life, the surroundings, the occupation itself of the dentist seem to develop healthfully this quality, and therefore I feel justified in adding this as one of the possessions of the dentist. I offer this as the result of extended acquaintance with, and large observation of dentists in this country and in Europe for more than a quarter of a century. Will any one deny it?

There are other items in the possession of dentists which as virtues are frequently grouped together, and which I can here group in the same way. They are *Faith*, *Hope and Charity*. *Faith*, the dentist certainly has, in the sense that he has faith in his efforts, in his influence, in his work, in the durability of his operations, etc. *Hope*, he must have to enable him to plod on and on, from day to day, in a narrow circle from early morn till night, and then again from morn till night, and still again and again, and again with a smiling face and a cheerful brow, a shining enthusiasm as wonderful as it is pleasant to see. For evidence, look about you to-day and observe the men of three score years, and then of three score years and ten—listen to their discussions—observe the keen knowledge of their interest in every thing that is new in dentistry (McK). Isn't it *hope* that fills their hearts? Hope that things may be brighter and easier in the future than they are now?

Charity—There are two kinds of charity—one the kind which,

" Gently views thy brother man,
Still gentler sister, woman," etc.,

And the other is expressed in the motto, " To sweet charity," and means giving.

It is the latter kind that the dentist has an abundance of. He gives his life, his best efforts, his time, his work, to his patients and thinks but little about the recompense. He is the charitable man in his daily life and work, *par excellence.* Many a man whose time is easily worth from ten to twenty dollars per hour, for *every* hour that he is in activity, gives hundreds of hours a year away to his friends, his relations and other people who could well afford to pay him for his time. He does it because of his innate *charity*. *Mercy* is an attribute of the dentist. Pointed at as he often is, as he walks the street as the " man who hurts " —surrounded as he is daily in his office with sharp cutting implements of torture—skilled as he is in the use of them—what man, if he were not filled to the brim with *mercy* would not *sometimes* give way to temper and revenge and hate, and destroy the patients who so often and yet so unwittingly insult him, and suspect his honesty, accuse him of meannesses, etc., etc., " Doctor, please don't make any holes in my teeth," archly cries the young married woman—" Is this poison which you have put in my tooth,"—" All the fillings which you put in have fallen out," —" My teeth never gave me any trouble till you filled them with the engine." Yet I say these people still live, and it is proof of the possession of this quality of mercy on the part of the dentist.

There are other items which could be added, but I did not promise your committee to write a treatise on the subject. I think I have mentioned enough points to satisfy you of the abounding wealth of the dentist.

